# Extract of *Adenanthera* *pavonina* L. seed reduces development of diabetic nephropathy in streptozotocin-induced diabetic rats

**Published:** 2012

**Authors:** Ramdas Pandhare, Balakrishnan Sangameswaran

**Affiliations:** 1 *Department of Pharmacology, MES College of Pharmacy, Sonai, Newasa, Ahmednagar,Maharashtra-414105, India*; 2 *Principal and Director Research Adesh Institute of Pharmacy and Biomedical Sciences Bathinda, Punjab, India*; 3 *Research Scholar, Department of Pharmacy, Suresh Gyan Vihar University, Jaipur, Rajasthan, India*

**Keywords:** *Adenanthera pavonina*, Albuminurea, Diabetic nephropathy, HbA1c, Proteinurea

## Abstract

**Objective:** The aim of the present study was to investigate the renal protective effect of *Adenanthera pavonina* (*A. pavonina*) seed aqueous extract (APSAE), in streptozotocin (STZ)-induced diabetic rats.

**Materials and Methods:** The renal protective effect of *A. pavonina* seed aqueous extract (APSAE) was studied in STZ-induced diabetic rats. APSAE (50, 100 and 200 mg/kg per day) was given daily to diabetic rats for 13 weeks. Blood glucose, serum parameters such as albumin, creatinine, total protein, urea, lipid profile, glycated haemoglobin (HbA1c), and urine parameters such as urine protein and albumin were examined. Kidney histopathology was also done.

**Results**: After 13 weeks of treatment, in STZ-induced diabetic rats, severe hyperglycemia was developed, with marked increase in proteinuria and albuminuria. However, APSAE treatment significantly reduced proteinuria, albuminuria, lipid levels, and HbA1c deposition in diabetic rats.

**Conclusion**: These results suggested that APSAE has reduced development of diabetic nephropathy in streptozotocin-induced diabetic rats and could have beneficial effect in reducing the progression of diabetic nephropathy.

## Introduction

Diabetic nephropathy is an important complication of both type 1 and type 2 diabetic mellitus (white et al., 2000). The clinical hallmarks of diabetic nephropathy include progressive albuminuria followed by a gradual decline in renal function. Glomerular basement thickening and mesangial expansion have been identified as pathological precursors of these clinical changes (Mauer et al., 1984). The loss of glomerular podocytes precedes and predicts the onset of clinical nephropathy and may be an early pathological manifestation of diabetic nephropathy (Pagtalunan et al., 1997; Meyer et al., 1999). Podocytes are one of the important ingredients of filtration barrier which have special cytobiological characteristic and physiological function. The injury of podocytes can unavoidably lead to the occurrence of proteinuria (Zang et al., 2007). Advanced glycation end products (AGEs) are a complex, heterogeneous, and sugar derived irreversible protein modifications that have been implicated in the pathogenesis of diabetic complications (Brownlee, 1995; Singh et al., 2001). The irreversible formation of AGEs affects proteins and lipids, such as haemoglobin, collagen and lipoprotein, and causes damage to the kidney, eyes, and blood vessels (Brownlee, 2005). The levels of AGEs are much higher in patients with diabetes. Moreover, it was reported that AGEs induce apoptosis of murine cultured podocytes. AGEs have been proposed for being the potential causative factor of podocyte damage (Chuang et al., 2007). Some medicinal herbs have also been used widely for the treatment of diabetes and diabetic complications for hundreds of years (Alarcon et al., 1998; Li et al., 2004). In the past few years, many herbal extracts have been screened for possible AGEs inhibitory effects in vitro.

*Adenanthera pavonina *Linn. (Family: Leguminosae-Mimosaceae), is a deciduous tree, 18-24 m tall, bole erect and 60 cm in diameter (Bouquet et al.,1974). Many species of *Adenanthera*, including *A. pavonina*, have been used as traditional herbal medicine against a variety of diseases. The plant is reported to have a wide range of biological activities, such as astringent and styptic (used in diarrhoea, stomach haemorrhage, haematuria) and anti-inflammatory (in rheumatic affections, gout) actions (Khare, 2007). Seeds are anticephalgic and also used for the treatment of paralysis.

 The seed contains an anti-inflammatory active principle, O-acetylethanolamine. The leaves contain octacosanol, dulcitol, glucosides of betasitosterol, and stigmasterol. The bark contains stigmasterol glucoside (Khare, 2007). Traditionally, the ground seed is widely used for the treatment of various human ailments such as treatment of boils, inflammation, blood disorders, arthritis, rheumatism, cholera, paralysis, epilepsy, convulsion, spasm, and indigestion (Burkill, 1966; Balogun et al., 2004). Phytochemically, the seed and its pod contain glycosides, saponins, and steroids (Howes, 1974; Yadav et al., 1976). A new five-membered lactone ring compound, pavonin, was isolated from the methanol soluble part of *A. pavonina *(Muhammad et al., 2005). Oil extracted from the seed has been reported to have membrane stabilizing activity by reducing lytic effect on erythrocytes, exhibited by many intravenous drugs (Anna et al., 2007). The methanol seed extract has also been reported to demonstrate anti-inflammatory and analgesic activities (Olajide et al., 2004). However, the effect of this herb on diabetes and diabetic complications is unclear. Therefore, in this study, we examined the renal protective effect of *A. pavonina *seed aqueous extract in STZ-induced diabetic rats.

## Materials and Methods

**Collection of plant material**

Seeds of *A. pavonina* were collected during March 2009 from the Mahatma Phule Krishi Vidyapeeth, Rahuri, Maharashtra, India. The leaves were identified by Dr. P.G. Diwakar, Joint Director, Botanical Survey of India, Pune. A voucher specimen (BSI/WRC/Tech/2010/463) has been kept in herbarium, Botanical Survey of India, Pune Maharashtra. These seeds were powdered and the powder was used for the extraction preparation. 

**Chemicals**

Streptozotocin (STZ) was purchased from Sigma chemical company, Banglore. All other chemicals used in the experiments were purchased locally (Merck and S. D. fine Chemicals) and were of analytical grade.

**Preparation of aqueous extract**

The powdered seed material was macerated with distilled water for 48 h at room temperature with occasional stirring. It was then filtered through Whatmann filter paper. The filtrate was air dried and stored in refrigerator for further use as APSAE (*Adenanthera pavonina* seed aqueous extract). The yield of the extract was 2.5% (w/w). During experiment the crude extract was diluted with distilled water just before oral administration to animals.

**Induction of diabetes**

Diabetes was induced in male Wistar albino rats aged 2–3 months (180–200 g body weight) by intraperitoneal administration of STZ (single dose of 55 mg/kg BW) dissolved in freshly prepared 0.01 M citrate buffer, pH 4.5 (Gupta et al., 2004). After 72 h, rats with marked hyperglycemia (fasting blood glucose≥250 mg/dl) were selected and used for the study. All the animals were allowed free access to tap water and pellet diet and maintained at room temperature in plastic cages, as per the guidelines of institutional animal ethics committee.

**Experimental design**

To investigate the effects of APSAE, the animals were divided into six groups each consisting of six animals as Group 1: Untreated normal rats, Group 2: Untreated diabetic rats, Group 3: Diabetic rats treated with glibenclamide at 0.25 mg/kg BW, Group 4: Diabetic rats treated with APSAE at 50 mg/kg BW, Group 5: Diabetic rats treated with APSAE at 100 mg/kg BW, Group 6: Diabetic rats treated with APSAE at 200 mg/kg BW 

After overnight fasting, *A. pavonina *seed aqueous extract suspended in distilled water was fed to the group 4, 5, and 6 rats by gastric intubation using a force feeding needle. Group 1 and 2 rats were fed with water alone and group 3 rats were fed with standard drug Glibenclamide daily orally for 13 weeks. 

**Metabolic and morphological analysis**

After 13 weeks period, blood samples were collected from the tail vein after 16 h fast and blood glucose estimation was carried out by glucose oxidase–peroxidase method (Kesari et al., 2005). HbA1c was estimated by the method of Eross (Eross et al., 1984). The estimation of serum lipids was carried out by the method of Folch (Folch et al., 1957). Estimation of serum cholesterol was carried out by the method of Zlatkis (Zlatkis et al., 1953). Serum triglycerides were estimated by the method of Foster (Foster et al., 1973) and HDL cholesterol was estimated by the method of Burstein (Burstein et al., 1970). The VLDL cholesterol was calculated using the formula TG/5 mg/dl (Burstein et al., 1970). The serum LDL cholesterol was estimated by the method of Friedwald (Friedwald et al., 1972). Similarly, serum parameters such as albumin, creatinine, urea, and total protein were also estimated. Individual rats were placed in metabolic cages to obtain 24-h urine collections, and urinary protein, albumin, and glucose excretion levels were measured.

**Immunohistochemical and immunofluorescent staining**

At 13 weeks, after a 24-h fast, the kidneys of all animals were collected for histopathological examination. In brief, the kidneys were preserved in 4% paraformaldehyde at room temperature for 24 h, embedded in paraffin, and sectioned (3 μm). Paraffin sections were deparaffinized, hydrated with water, and stained with periodic acid Schiff (PAS) reagent and haematoxylin as a counterstain. Sections were observed under light microscope (450X) for number of mesangial cells, matrix of glomeruli, and hyaline thickening of arterioles (Sohn et al., 2007). 

**Statistical analysis**

All values were expressed as Mean±SEM. The statistical analysis of the difference was carried out by using one way analysis of varience (ANOVA) followed by Dunnette’s multiple comparison test and significant level was assumed at p<0.05.

## Results

**Body weight and serum biochemical parameters**

At the end of 13 weeks treatment with APSAE and Glibenclamide, the body weight of normal, APSAE, and glibenclamide-treated rats were significantly increased compared with the diabetic control group (Table 1). Similarly, animals treated with APSAE and glibenclamide showed a significant decrease in blood glucose and HbA1c level as compared with diabetic control rats (Table 1).

**Assessment of long-term effect of APSAE on lipid profiles in normal and STZ-induced diabetic rats**

In STZ-induced diabetic rats, after 13 weeks of treatment, the effect of aqueous extract on serum triglycerides (TG), total cholesterol, LDL, VLDL, and HDL cholesterol in all the experimental groups of rats were studied. The serum triglycerides, total cholesterol, LDL, and VLDL cholesterol levels were significantly increased, while the HDL cholesterol levels decreased in the diabetic control rats when compared with normal rats. Treatment of the diabetic rats with APSAE and glibenclamide caused a significant reduction in the serum triglycerides, total LDL, and VLDL cholesterol with a significant increase in HDL cholesterol levels when compared with diabetic control rats (Table 2).

**Assessment of long-term effect of APSAE on serum parameters in normal and STZ-induced diabetic rats**

In STZ-induced diabetic rats, after 13 weeks of treatment, the effect of aqueous extract on serum albumin, creatinine, urea, and total protein in all the experimental groups of rats were studied. The serum albumin, creatinine, urea, and total protein levels were significantly higher in diabetic control rats compared with those in normal rats. However, treatment of the diabetic rats with APSAE produced a significant reduction in serum albumin, creatinine, urea, and total protein levels when compared with diabetic control rats (Table 3). 

**Assessment of long-term effect of APSAE on urine parameters in normal and STZ-induced diabetic rats**

In STZ-induced diabetic rats, after 13 weeks of treatment, the effect of aqueous extract on urine glucose, albumin, and total protein in all the experimental groups of rats were studied. The urine glucose, albumin, and total protein levels were significantly higher in diabetic control rats compared with those in normal rats. Treatment of the diabetic rats with APSAE produced a significant reduction in the albumin, creatinine, urea and total protein levels when compared with diabetic control rats (Table 4).

**Immunohistochemical and immunofluorescent study**

**Figure 1 (A-F):**
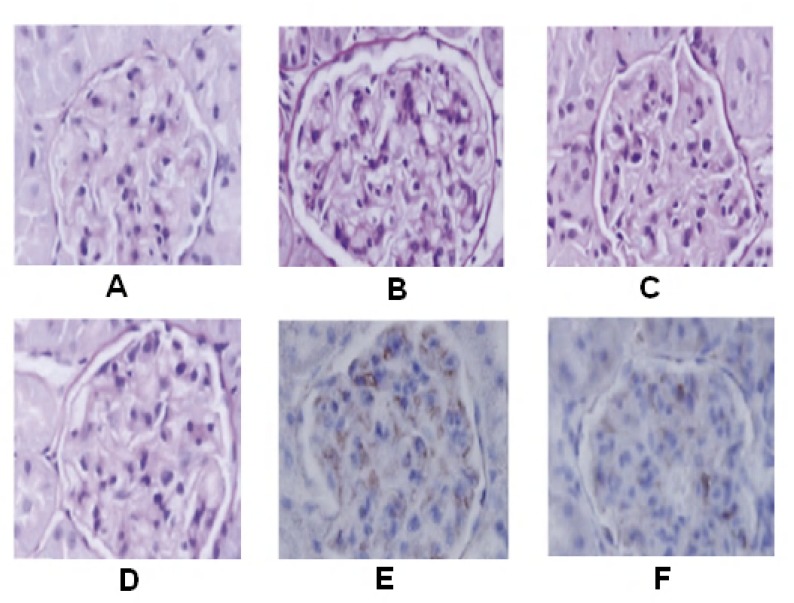
Histopathological changes in the kidney where, images **A-F** shows transverse-section of kidney of normal, diabetic control, diabetic treated with APSAE (50, 100 and 200 mg/kg) and glibenclamide pretreated groups, respectively (A-F).

Diabetic nephropathy resulted in significant histopathological changes assessed in transverses section of the kidney. In transverse section nerve derangement, axonal swelling, increase in number of mesangial cells, matrix of glomeruli, and hyaline thickening of arterioles were also noted. Administration of the APSAE (50, 100, and 200 mg/kg p.o.) significantly attenuated diabetes induced fiber derangement, swelling of nerve fibers, and increase in number of mesangial cells as a marker of histopathological alterations when compared with diabetic control group (Figure 1a-f). Microscopic examinations were performed under 450X light microcopy, scale bar 35 μm. 

**Table 1:** Effect of *Adenanthera pavonina *seed aqueous extract on blood glucose, glycosylated Hb and body weights of normal and diabetic rats

**Group Treatment** **(n=6)**	**Blood glucose (mg/dl)**	**HbA1c ** **mg/gm Hb**	**Change in body weight (gm)**
**I - Normal control**	103.17±4.57	6.45±0.17	186.17±1.70
**II - Diabetic control**	413.33±2.80	10.83±0.11	142.50±1.25
**III- Diabetic + glibenclamide (0.25mg/kg)**	106.33±1.28**	7.50±0.005**	170.17±1.70 **
**IV- Diabetic + APSAE (50mg/kg)**	125.83±0.60*	8.40±0.38**	156.83±1.77**
**V- Diabetic + APSAE (100mg/kg)**	118.00±0.57**	8.16±0.04**	164.33±1.30**
**VI- Diabetic + APSAE (200mg/kg)**	113.00±1.03**	7.80±0.07**	170.67±1.20**

Values are Mean±SEM, n=6, when compared with diabetic control by using one way ANOVA followed by Dunnette’s multiple comparison test.

* p<0.05,

** p<0.01.

**Table 2:** Effect of *Adenanthera pavonina *seed aqueous extract on serum lipid profile of normal and diabetic rats.

**Group (n=6)**	**Body Cholesterol **	**LDL**	**HDL** **in mg/dl**	**VLDL**	**Triglycerides **
**I **	66.83±0.47	23.33±0.42	11.50±0.42	16.66±0.30	66.00±0.57
**II **	94.00±0.73	95.50±0.76	8.00±0.51	23.00±0.36	113.17±0.60
**III**	69.66±0.49**	36.83±0.60 **	12.16±0.30 **	17.83±0.65 **	75.50±0.76 **
**IV**	81.16±0.47*	53.16±0.47 **	12.33±0.33 **	20.33±0.42*	82.50±0.76 **
**V**	77.00±0.57 **	46.66±0.49 **	13.33±0.33 **	18.83±0.40 **	79.50±0.78 **
**VI**	73.50±0.42 **	38.83±0.60 **	13.66±0.42 **	18.66±0.21 **	77.50±0.38 **

Values are Mean±SEM, n=6, when compared with diabetic control by using one way ANOVA followed by Dunnette’s multiple comparison test.

* p<0.05,

** p<0.01.

**Table 3:** Effects of *Adenanthera pavonina *seed aqueous extract on serum parameters of normal and diabetic rats.

**Group Treatment** **(n=6)**	**Albumin ** **(mg/dl)**	**Creatinine (mg/dl) **	**Urea(mg/dl)**	**Total protein(mg/dl)**
**I - Normal control**	3.26±.004	0.58±0.05	29.66±0.88	7.26±0.12
**II - Diabetic control**	2.40±0.12	2.56±0.20	69.00±1.78	4.98±0.13
**III- Diabetic + glibenclamide (0.25mg/kg)**	3.16±0.01**	0.93±0.07 **	36.66±0.88 **	6.96±0.07 **
**IV- Diabetic + APSAE (50mg/kg)**	2.75±0.08*	1.55±0.07*	52.16±1.35*	5.81±0.21*
**V- Diabetic + APSAE (100mg/kg)**	2.98±0.02 **	1.20±0.05 **	40.33±0.88 **	6.26±0.08 **
**VI- Diabetic + APSAE (200mg/kg)**	3.10±0.08 **	1.07±0.04 **	39.16±0.47 **	6.75±0.07 **

Values are Mean±SEM, n=6, when compared with diabetic control by using one way ANOVA followed by Dunnette’s multiple comparison test.

* p<0.05,

** p<0.01.

Figures** A** and **F** shows normal fiber arrangement with mesangial cells. Figure **B** shows nerve derangement, axonal swelling, increase in number of mesangial cells, matrix of glomeruli, and hyaline thickening of arterioles. Figure **C, D,** and **E** shows attenuation of STZ-induced nerve derangement, axonal swelling and increase in number of mesangial cells and matrix of glomeruli. Microscopic examinations were performed under 450X light microcopy, scale bar 35 μm.

**Table 4:** Effect of *Adenanthera pavonina *seed aqueous extract on Urine parameters of normal and diabetic rats.

**Total protein** **(mg/dl)**	**Albumin** **(mg/dl)**	**Glucose ** **(mg/dl)**	**Group Treatment** **(n=6)**
6.28±0.14	2.15±0.07	0.23±.004	**I - Normal control**
25.81±0.31	24.10±0.69	3.63±0.13	**II - Diabetic control**
6.95±0.26**	4.95±0.22**	1.01±0.08**	**III-Diabetic + glibenclamide (0.25mg/kg)**
9.91±0.14 **	8.66±0.12 **	1.95±0.10*	**IV- Diabetic + APSAE (50mg/kg)**
8.95±0.12 **	7.58±0.14 **	1.68±0.10 **	**V- Diabetic + APSAE (100mg/kg)**
7.66±0.11 **	6.30±0.15 **	1.53±0.08 **	**VI- Diabetic + APSAE (200mg/kg)**

Values are Mean±SEM, n=6, when compared with diabetic control by using one way ANOVA followed by Dunnette’s multiple comparison test.

* p<0.05,

** p<0.01.

## Discussion

Diabetic nephropathy is one of the most common complications of diabetes and is characterized by increased urinary protein and loss of renal function. A number of studies have now definitely proven that improved metabolic control that achieves near-normoglycemia can significantly decrease the development and progression of diabetic nephropathy (Mogensen, 1984; Lee et al., 1995; Park et al., 1998). The metabolic factors such as AGEs, sorbitol beyond blood glucose level are also implicated in the pathogenesis of diabetic nephropathy (Schrijvers et al., 2004). Traditional plant remedies have been used for centuries in the treatment of diabetes (Akhtar et al., 1984; Kesari et al., 2005; Kesari et al., 2007; Rai et al., 2007), but only few of these plants have been scientifically evaluated. Therefore, we have investigated the effect of APSAE on glycemic and renal protection in STZ-induced diabetic rats. *A. pavonina* seed aqueous extract showed a dose dependent effect on fasting blood glucose in diabetic rats. The capacity of APSAE to decrease the elevated blood glucose to normal level is an essential trigger for the liver to revert to its normal homeostasis during experimental diabetes. The possible mechanism by which APSAE exerts its hypoglycemic action in diabetic rats may be due to potentiating the insulin release, since the percentage of reduction in blood glucose levels was considerable. In uncontrolled or poorly controlled diabetes, there is an increased glycosylation of a number of proteins including haemoglobin and crystalline of lens (Alberti, 1982). HbA1c was found to increase in patients with diabetes mellitus and the amount of increase was directly proportional to the fasting blood glucose levels (Pari et al., 2002). 

Therefore, measurement of HbA1c is supposed to be a very sensitive index for glycemic control. Treatment with APSAE showed a significant decrease in the glycated haemoglobin levels, which could be due to an improvement in insulin secretion. Induction of diabetes with STZ is associated with the characteristic loss of body weight, which is due to increased muscle wasting (Swanston et al., 1990) and due to loss of tissue proteins (Chatterjea et al., 1976). Diabetic rats treated with the APSAE showed an increase in body weight when compared with the diabetic control rats which may be due to its protective effect in controlling muscle wasting, i.e., reversal of gluconeogenesis and also the improvement in glycemic control.

The results from the current study showed that APSAE reduces the development of diabetic nephropathy by increasing serum parameters such as albumin and total protein, but a reduction in serum creatinine and urea as well as urine albumin and total protein in treated rats as compared with diabetic control rats. Hence, current study confirmed that APSAE-treated diabetic rats showed significant improvement in renal functions such as proteinuria and albuminuria.Treatment of the diabetic rats with APSAE and glibenclamide caused a significant reduction in the serum triglycerides, LDL and VLDL cholesterol with a significant increase in HDL cholesterol levels when compared with diabetic control rats. Administration of the APSAE (50, 100 and 200 mg/kg p.o.) significantly attenuated diabetes induced fiber derangement, swelling of nerve fiber, and increase in number of mesangial cells as a marker of histopathological alterations when compared with diabetic control group. In conclusion, the present data clearly demonstrate that administration of APSAE reduces metabolic factors influencing diabetic nephropathy such as blood glucose, albumin, total protein creatinine and HbA1c in experimental diabetes. Taking together these results indicate that, APSAE has therapeutic or preventive effects on several pharmacological targets in the complicated pathological mechanism of diabetic nephropathy. Thus, it is worthwhile to be further investigated for its potential pharmacological effects in diabetic nephropathy.
